# Ultra-Short-Term Wind Farm Power Prediction Considering Correlation of Wind Power Fluctuation

**DOI:** 10.3390/s24206538

**Published:** 2024-10-10

**Authors:** Chuandong Li, Minghui Zhang, Yi Zhang, Ziyuan Yi, Huaqing Niu

**Affiliations:** 1 College of Mechanical and Electrical Engineering, Fujian Agriculture and Forestry University, Fuzhou 350100, China; lichuandong@126.com; 2 Electric Power Research Institute of State Grid Fujian Electric Power Company, Fuzhou 350003, China; zhangyi@fzu.edu.cn (M.Z.); yiziyuan2019@126.com (Z.Y.); 3 College of Electrical Engineering and Automation, Fuzhou University, Fuzhou 350108, China

**Keywords:** adjacent wind farms, ultra-short-term output prediction, spatial–temporal correlation, prior information period, variational Bayesian model

## Abstract

Accurate ultra-short-term power prediction for wind farms is challenging under rapid wind speed fluctuations, complicating production planning and power balancing. This paper proposes a new method considering spatial and temporal correlations of wind fluctuations among adjacent wind farms. The method first calculates the time difference between power fluctuations based on wind speed, direction, and relative positions, determining the prior information period. The variational Bayesian model is then used to extract implicit relationships between power fluctuations of adjacent wind farms, enabling power prediction during the prior information period. Finally, the non-prior information period is predicted to complete the ultra-short-term power prediction. Using measured data from three wind farms in Fujian Province, compared to other models, the method demonstrates improved accuracy by effectively leveraging the power fluctuation characteristics of adjacent wind farms, and it has a certain amount of generalizability.

## 1. Introduction

As an intermittent power source, wind power’s inherent volatility brings great challenges to the real-time power balance of the power system. Accurate prediction of wind farm output is the basis of real-time supply and demand balance of the power grid and the prerequisite for safe and economic operation of the power grid [1,2].

At present, wind power prediction is a research focus in new power systems, which can be roughly divided into two categories: one is the physical method based on mechanistic portrayal and mathematical abstraction [3]; the other is the data-driven method based on data mining laws [4,5]. With the deepening and development of related research, the two methods have also shown a trend of integration. The ultra-short-term prediction model driven by the historical power data of wind farms gradually takes meteorological data into account [6]. For example, in Gu B et al. [7,8], by mining the correlation between meteorology and power in the historical data of wind farms, the meteorological prediction based on mechanistic construction is gradually integrated into the ultra-short-term power prediction of wind farms, which is used to improve the accuracy of power prediction. From the current research results, the research direction of ultra-short-term wind farm power prediction mainly focuses on how to effectively consider more power influencing factors, how to select efficient rule mining methods to improve the accuracy of prediction, and the convenience of model use.

With the intensive development of wind power resources, wind farms are increasingly showing spatial agglomeration [9]. The power fluctuations of wind farms in the same region are highly correlated. Many researchers have applied this factor to the power prediction of wind farms, thereby effectively improving the accuracy of power prediction [10,11]. Zhao Z et al. [12] simplified the relationship between the spatial relative position and the power of the wind farm into a linear relationship and used the vector autoregressive model to predict the power of the wind farm. The method presupposes the functional relationship between the power and the spatial position of the wind farm, and then uses the data-driven method to fit. Although the modeling problem of how the spatial position of the wind farm influences its power has been initially solved, the actual power of the wind farm is affected by many factors and contains complex nonlinear components. The above method is too hypothetical and limited. Therefore, the recent related research mainly focuses on how to mine implicit complex nonlinear relationship.

Zhang H et al. [13] used IDMDN to learn the nonlinear mapping relationship between NWP data and prediction results of multi-wind farms. Dong X et al. [14] used STCN to learn the characteristics of different scales and predict the power of wind farms. The nonlinear correlation between the power of different wind farms is implicitly fitted to improve the accuracy of power prediction. The above methods realize the extraction of the nonlinear correlation between the geographical location and the power of the wind farm to some extent.

However, the following problems still exist: (1) Due to the inherent ‘black box’ characteristics of machine learning algorithms, the model has poor generalization performance for scenarios that have not appeared in historical data. (2) Before the model training, the existing methods do not effectively simplify the relationship between the influencing factors based on physical laws such as meteorological processes, and cannot reduce the fitting function space, making model training difficult. (3) In the case of large fluctuations in wind farm power, it is impossible to capture the most effective key factors for predicting power through intuitive physical meaning. It is still difficult to effectively apply in the ultra-short-term power prediction of actual wind farms.

In view of the above research status, based on the spatial and temporal correlation of power fluctuations between wind farms, we propose a new method for ultra-short-term power prediction that considers the influence of power fluctuations of adjacent wind farms. The primary contributions here include the following:

(1) Based on the relative position of the wind farms and the real-time meteorological data, the adjacent wind farm with the power fluctuations prior to the target wind farm and the nearest distance is selected as the first wind farm. Its power fluctuations are considered as the prior information.

(2) The time difference between the power fluctuations of two wind farms caused by the same wind speed is estimated. The ultra-short-term prediction period of the target wind farm (4 h selected in this paper) is dynamically divided into prior information period and a non-prior information period.

(3) In the prior information period, the variational Bayesian model is used to comprehensively consider the relationship between historical power.

(4) Compared with the existing prediction methods, this method exhibits higher prediction accuracy and generalization, which can provide an important reference for the risk assessment of the power system.

## 2. Spatial–Temporal Correlation Analysis

In the ultra-short-term prediction of wind farm power, the amount of its power has a strong correlation with the strength of wind speed [15]. In the plains, ignoring the effect of topography, when the wind blows through an area, the power fluctuation caused by this gust should be similar for the different wind farms in the area. Due to the movement of the wind taking a certain time, a time difference results between the similar power fluctuations of wind farms [16]. Using this time difference can provide effective prior information for the power prediction of the target wind farm.

### 2.1. Selection of Prior Information Sources

Due to the gradual clustering of the spatial distribution of wind farms, there are multiple wind farms that may provide prior information for the target wind farm under the same wind direction. Therefore, how to select the prior information becomes a problem to be solved. We simplify this problem by adopting the approach of selecting the most directly associated adjacent wind farms.

Considering the way in which wind affects wind farm power, we presuppose that the wind farm closest to the target wind farm in the dominant wind direction is most likely to directly affect the target wind farm, and therefore this wind farm provides the most effective prior information. Based on this preset, we select the wind farm used for extracting the prior information based on the distance between the adjacent wind farms and the target wind farm, and the angle between the relative positional connecting line and the wind direction. This is shown in Formula (1):

(1) $$W(i)=\arg\min(d(i)+\cos(\theta_{P}(i)-\theta_{w}))$$

where *θ_w_* is the wind direction at the prediction time; **W**(*i*) is the *i*-th adjacent wind farm; ***θ_p_***(*i*) is the angle between the north direction and the line segment connecting the *i*-th adjacent wind farm starting from the target wind farm; and ***d***(*i*) is the distance from the *i*-th adjacent wind farm to the target wind farm after normalizing the distance from the adjacent wind farms to the target wind farm.

After identifying the adjacent wind farm that can provide the prior information, which we refer to as the first wind farm of the target wind farm, the validity of the prior information is verified later in Section 5.2.

### 2.2. Dynamically Divide the Prediction Period

At the macroscale, adjacent wind farms usually have similar main wind directions, so that in most cases, it is possible to find wind farms that experience the wind power fluctuations earlier than the target wind farm. We attempt to improve the accuracy of ultra-short-term prediction for wind farms with large power fluctuations by effectively identifying this physical process. Therefore, after identifying the first wind farm that provides the prior information, it is necessary to calculate the time difference *T* that exists in the fluctuation-related power between it and the target wind farm.

With different wind speeds and directions, *T* will also change. Therefore, at each prediction time, *T* needs to be calculated based on the relative geographical location between wind farms and real-time wind speed and direction, as shown in Formula (2):

(2) $$T=\frac{d}{v\times \cos(\theta_{P}-\theta_{w})}$$

where *d* is the distance between the target wind farm and the first wind farm; *θ_p_* is the angle between the north direction and the line segment connecting the first wind farm starting from the target wind farm; and *v* is the wind speed at the prediction time.

**Figure 1:**
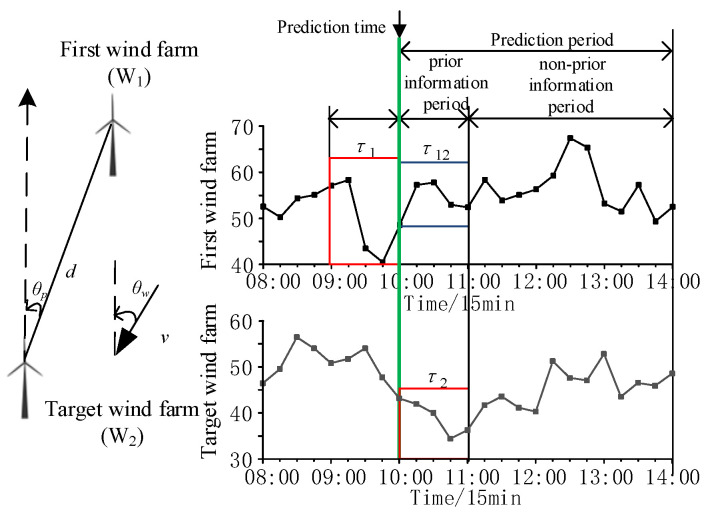
The relative geographical location and prediction period diagram of wind farms.

The *T* before the prediction time of the first wind farm is *τ*_1_, and the *T* after the prediction time of the target wind farm is *τ*_2_, as shown in Figure 1. Because the fluctuation of power in *τ*_1_ is related to *τ*_2_ and the power in *τ*_1_ is known, it can provide prior information for the fluctuation of power in *τ*_2_. The correlation between power in *τ*_1_ and *τ*_2_ is verified in Section 5.2.

According to *T* calculated at each prediction time, the prediction period of the target wind farm is dynamically divided into a prior information period (*τ*_2_) and a non-prior information period, as shown in Figure 1. The calculation formula for the number of power points *h* in the prior information period is shown in Formula (3):

(3) $$h=\frac{T}{T_{0}}$$

where *T*_0_ is the sampling time interval of wind farm power.

We take into account both wind speed and wind direction around the wind farms when calculating *T*. The basic principle is to estimate the length of time for which the power fluctuation occurs in the first wind farm ahead of the target wind farm by using the information on wind speed and wind direction as well as the geographic location of the wind farm. Therefore, the calculation of *T* is premised on the following two aspects: firstly, the wind direction of the wind farms in the region basically does not change much within a few hours; secondly, the wind speed attenuation is not significant when the wind propagates between adjacent wind farms.

Through the selection of the first wind farm in each prediction period and the changes of *T* and *h*, the most direct prior information can be found for the target wind farm, and the changing spatiotemporal correlation between the wind farms can be fully expressed.

## 3. Basic Theories

There are two main factors affecting the power of the target wind farm with prior information: (1) the historical power data of the target wind farm; (2) the prior information provided by the first wind farm. To extract the influence of each factor on the power, we use the probability distribution sequence of the time series to describe the variation range of the power under the influence of each individual factor. The variational Bayesian model is used to fit the relationship between the combined influence of both factors and the predicted power. This approach allows for us to predict the power during the prior information period.

### 3.1. Probability Distribution Sequence Considering Only Single Factor Influence

In the prior information period, the prediction results of the two influencing factors are expressed by the probability distribution sequence ***P_a_*** and Δ***P_f_***. ***P_a_*** is the power sequence that only considers the influence of the historical power of the target wind farm, while Δ***P_f_*** is the power fluctuation sequence that only considers the influence of prior information. The reason why Δ***P_f_*** chooses the power fluctuation sequence instead of the power sequence is that it is difficult to classify the same power fluctuations in the first wind farm using the power sequence. This is not conducive to the calculation of the probability distribution corresponding to the target wind farm variation under the same conditions.

To reflect the possible variation range of the target wind farm output under the action of a single factor, this paper uses normal distribution to describe each point in the two probability distribution sequences of ***P_a_*** and Δ***P_f_***, as shown in Formula (4).

(4) $$\left\{\begin{array}{l} P_{a}=\Pi_{i=1}^{h}N(\mu_{P_{a},i},\sigma_{P_{a},i})\\ \Delta P_{f}=\Pi_{i=1}^{h}N(\mu_{\Delta P_{f},i},\sigma_{\Delta P_{f},i}) \end{array}\right.$$

where $\mu_{P_{a},i}$ and $\sigma_{P_{a},i}$ are the mean and variance of the *i*-th point in ***P_a_***; $\mu_{\Delta P_{f},i}$ and $\sigma_{\Delta P_{f},i}$ are the mean and variance of the *i*-th point in Δ***P_f_***.

When calculating ***P_a_***, we use LSTM to fit the correlation between the power sequence of the prior information period and the historical power sequence. By inputting the power sequence before the prediction time of the target wind farm into the LSTM, the average value $\mu_{P_{a}}$ and variance sequence $\sigma_{P_{a}}$ of the power in the prior information period are obtained.

In the prior information period of the target wind farm, the trend of change of prior information and power is similar, but there are still differences that are difficult to analyze. In the historical data, according to the magnitude of the prior information power fluctuations, the power fluctuations of the prior information period are segmented and counted. The distribution of power fluctuations in each section is obtained. Finally, at the prediction time, based on the obtained prior information, the distribution of each point in Δ***P_f_*** is determined. The specific practices are as follows:

(1) In the historical data of the target wind farm, each prediction period is divided. Find the corresponding prior information for each prior information period. In chronological order, the order of time, the variation in prior information, and the variation in power in the prior information period are composed into two sequences, as shown in Formula (5).

(5) $$\left\{\begin{array}{l} \Delta P_{Fir}=[\Delta p_{1,1,1},\cdots ,\Delta p_{1,1,h_{1}},\cdots ,\Delta p_{1,i,1},\cdots ,\Delta p_{1,i,h_{i}}]\\ \Delta P_{Aft}=[\Delta p_{2,1,1},\cdots ,\Delta p_{2,1,h_{1}},\cdots ,\Delta p_{2,i,1},\cdots ,\Delta p_{2,i,h_{i}}] \end{array}\right.$$

where Δ***P_Fir_*** is the variation sequence of prior information; Δ***P_Aft_*** is the variation sequence of power in the prior information period; *h_i_* is the number of power points of the prior information period in the *i*-th prediction period; $\Delta p_{1,i,h_{i}}$ and $\Delta p_{2,i,h_{i}}$ are the *i*-th power fluctuations of the prior information and the prior information period in the *i*-th prediction period.

(2) Each point in ΔPFir is sorted from small to large to obtain ΔP’Fir, which is divided into k intervals by quantile [17]. Using the corresponding relationship between the *i*-th point in Δ***P_Fir_*** and the *i*-th point in Δ***P_Aft_***, the points in Δ***P_Aft_*** are classified into the corresponding intervals. The mean and variance of Δ***P_Aft_*** in each interval are calculated, as shown in Formula (6):

(6) $$\left\{\begin{array}{l} \mu_{\Delta P_{Aft}}=[\mu_{1},\mu_{2},\cdots ,\mu_{k}]\\ \sigma_{\Delta P_{Aft}}=[\sigma_{1},\sigma_{2},\cdots ,\sigma_{k}] \end{array}\right.$$

where $\mu_{P_{a},i}$ and $\sigma_{P_{a},i}$ are the mean and variance sequences of Δ***P_Aft_***; *μ_k_* and *σ_k_* are the mean and variance of the power fluctuations in the target wind farm in the *k*th interval, respectively.

(3) The prior information is obtained at the prediction time, and the variation sequence is obtained. Through the interval corresponding to each point of the sequence, the $\mu_{\Delta P_{f}}$ and $\sigma_{\Delta P_{f}}$ of Δ***P_f_*** are determined. That is, when the *i*-th point of the prior information change sequence is in the *j*-th interval, the *i*-th point of $\mu_{\Delta P_{f}}$ and $\sigma_{\Delta P_{f}}$ in the prior information period is *μ_j_* and *σ_j_*, respectively.

In practical applications, due to insufficient historical data or other reasons, a certain amount of change in the prior information may fall outside the range of Δ***P_Fir_***. At this time, it will first be classified into the relatively closest interval, and the mean and variance of the interval are selected as the prediction results corresponding to the point. After the prediction is completed, the data in Δ***P_Fir_*** and Δ***P_Aft_*** are updated to make the contained range as comprehensive as possible.

### 3.2. Variational Bayesian Model Considering Two Factors Comprehensively

The calculation of ***P_a_*** and Δ***P_f_*** only considers a single influencing factor, while the actual power of the target wind farm is the result of the combined action of both factors. If only ***P_a_*** and Δ***P_f_*** are calculated, there will be a significant gap between the predicted power and the actual power. The variational Bayesian model, which is based on Bayesian theorem and variational inference, can optimize the probability distribution by minimizing the KL divergence and make it approximate the real distribution [18]. Therefore, we use KL divergence to characterize the distance between ***P_a_*** and Δ***P_f_*** and the real distribution and use the variational Bayesian model to modify ***P_a_*** and Δ***P_f_*** to make them continue approaching the real distribution. The target formula is shown in Formula (7).

(7) argminKL(q(ψ)||p(ψ|y))

where ***ψ*** = {Δ***P_f_*, *P_a_***} is the set of variables, *p*(***ψ***|***y***) is the real distribution of wind farm power under the combined action of two factors; ***y*** is the real value label of wind farm power corresponding to ***ψ***; *q*(***ψ***) is the probability distribution sequence under the respective action of two factors.

However, the traditional variational Bayesian model requires a complex optimization process when approaching the real distribution. It takes a long time and can easily lead to inaccurate approximate inference [19]. Deep neural networks can quickly learn complex mapping relationships. Therefore, we use them to fit the relationship between the two probability distribution sequences considering a single influencing factor and the real power sequence of the wind farm, and approximate ***P_a_*** and Δ***P_f_***.

**Figure 2:**
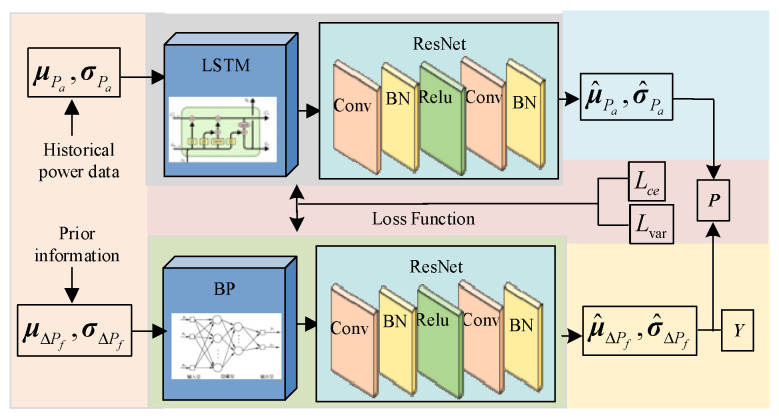
The variational Bayesian model based on a deep neural network.

BP has strong nonlinear fitting ability, while LSTM pays more attention to the dependencies in the data sequence and can learn the weights and biases at different time points. Therefore, we use BP and LSTM to approximate Δ***P_f_*** and ***P_a_*** in the deep inference model. The specific process is shown in Figure 2.

In Figure 2, after using the historical power data of the target wind farm and the prior information to obtain ***P_a_*** and Δ***P_f_***, the respective mean and variance sequences are input into the corresponding neural network. The neural network is combined with ResNets in CNN. The structure of the ResNets is ‘Conv+ BN+ ReLU+ Conv+ BN’, and two output channels are set to output the mean and variance after approximation. By using ***Y***, that is, the power of the change at the previous moment, the approximated power fluctuation sequences ${\hat{\mu }}_{\Delta P_{f}}$ and ${\hat{\sigma }}_{\Delta P_{f}}$ are restored to the power sequence. It is weighted with the approximated power sequences ${\hat{\mu }}_{P_{a}}$ and ${\hat{\sigma }}_{P_{a}}$, and the power prediction result of the prior information period ***P*** considering the two influencing factors is obtained.

In the variational Bayesian model, we use two deep neural networks to evaluate the deviation between the power prediction results and the actual power. To construct a reasonable loss function, we combine the cross-entropy loss and the variational loss obtained from the objective function to train the model. The cross-entropy loss is used to measure the difference between the true value and the power results of the two considering the influence of single factor, while the variational loss is used to measure the difference between the probability distribution of the power and the true distribution. The combination of the two loss functions can make the model more robust and can better deal with the situation with large uncertainty.

### 3.3. Construction of Loss Function

The wind power prediction output sequence ***P*** and the real value sequence ***P_r_*** are brought into Formula (7) for parameterization, and Formula (8) can be obtained.

(8) $$\begin{array}{l} \arg\min KL(q(\psi )||p(\psi ))-E_{q}[\log p(P,P_{r}|\psi )]\\ =\arg\min KL(q(\psi )||p(\psi ))-E_{q}[\log p(P|\psi )]-\\ E_{q}[\log p(P|P_{a})] \end{array}$$

The first and second terms are the variational loss *L*_var_; the third term is the cross-entropy loss *L*_ce_. If the optimization goal of the model is set to minimize Formula (8), it will lead to an increase in *L*_ce_. Therefore, we focus on the minimization of *L*_var_ and expand it to obtain Formula (9).

(9) $$\begin{array}{l} L_{\mathrm{var}}=KL(q(\psi )||p(\psi ))-E[\log p(P|\psi )]\\ =KL(q(\Delta P_{f})||p(\Delta P_{f}))+KL(q(P_{a})||p(P_{a}))-\\ E[\log p(P|P_{a},\Delta P_{f})] \end{array}$$

*L*_var_ can be summarized into three aspects: (1) the variational loss *L_p_* of the power curve, which represents the loss between the actual value and the predicted value of the power; (2) the loss of Δ***P_f_***, which is composed of mean loss $L_{{\hat{\mu }}_{\Delta P_{f}}}$ and variance loss $L_{{\hat{\sigma }}_{\Delta P_{f}}}$; and (3) the loss of ***P_a_***, which is composed of mean loss $L_{{\hat{\mu }}_{P_{a}}}$ and variance loss $L_{{\hat{\sigma }}_{P_{a}}}$. The specific calculation formula is as follows:

(10) $$L_{\mathrm{var}}=L_{P}+L_{{\hat{\mu }}_{\Delta P_{f}}}+L_{{\hat{\sigma }}_{\Delta P_{f}}}+L_{{\hat{\mu }}_{P_{a}}}+L_{{\hat{\sigma }}_{P_{a}}}$$

(11) $$\left\{\begin{array}{c} L_{P}=\frac{1}{2}||2P-(P_{a}+\Delta P_{f}+Y)||\\ L_{{\hat{\mu }}_{\Delta P_{f}}}=\frac{1}{2}\sum_{i=1}^{h}\sigma_{\Delta P_{f},i}{{\hat{\mu }}_{\Delta P_{f},i}}^{2}\\ L_{{\hat{\sigma }}_{\Delta P_{f}}}=\frac{1}{2}\sum_{i=1}^{h}(\sigma_{\Delta P_{f},i}{\hat{\sigma }}_{\Delta P_{f},i}^{2})-<I,\mathrm{In}({\hat{\sigma }}_{\Delta P_{f}}^{2})>\\ L_{{\hat{\mu }}_{P_{a}}}=\frac{1}{2}\sum_{i=1}^{h}\sigma_{P_{a},i}{\hat{\mu }}_{P_{a},i}^{2}\\ L_{{\hat{\sigma }}_{P_{a}}}=\frac{1}{2}\sum_{i=1}^{h}(\sigma_{P_{a},i},{\hat{\sigma }}_{P_{a},i}^{2})-<I,\mathrm{In}({\hat{\sigma }}_{P_{a}}^{2})> \end{array}\right.$$

where ${\hat{\mu }}_{\Delta P_{f},i}$ and ${\hat{\sigma }}_{\Delta P_{f},i}$ are the *i*-th point in ${\hat{\mu }}_{P_{a},i}$ and ${\hat{\sigma }}_{P_{a},i}$; ${\hat{\mu }}_{P_{a},i}$ and ${\hat{\sigma }}_{P_{a},i}$ are the *i*-th point in ${\hat{\mu }}_{P_{a}}$ and ${\hat{\sigma }}_{P_{a}}$.

Because we are carrying out ultra-short-term prediction, the prediction results of the interval are not significant. Therefore, the output channel of the residual block is set to 1, and the variational Bayesian model is simplified. Only the mean of the model is output, while the variance is used as the input feature of the model to assist in the approximation of the mean. The simplified variational loss *L*_var_ is shown in Formula (12):

(12) $$L_{\mathrm{var}}=L_{P}+L_{{\hat{\mu }}_{\Delta P_{f}}}+L_{{\hat{\mu }}_{P_{a}}}$$

Then, on the basis of minimizing *L*_var_, the loss function is constructed by the balance between *L*_ce_ and *L*_var_, as shown in Formula (13):

(13) $$\min L_{ce}+\lambda L_{\mathrm{var}}$$

where *λ* is the equilibrium weight, which we set to 50 after several attempts based on the calculus.

### 3.4. The Interpretability of Model

According to Zhang et al., the interpretability of the improved variational Bayesian model can be reflected in three dimensions [20]: (1) According to the number of power points that need to be predicted in the prior information period, different models are established. Active interpretability is reflected by selecting different models at each prediction time. (2) After obtaining the prediction results ***P_a_*** and Δ***P_f_*** that only consider the influence of a single factor, the balance of *L*_ce_ and *L*_var_ is used to approximate the real distribution. The prediction result of the two influencing factors is comprehensively considered. The interpretability focuses on the overall decision logic of the model, reflecting the global interpretability. (3) The physical meaning of variables in the variational Bayesian model is clear, enhancing the comprehensibility and interpretability of the model. For example, Δ***P_f_*** represents the prediction result of power fluctuations only considering the influence of prior information, and ***P_a_*** represents the prediction result of power only considering the influence of historical power of the target wind farm.

On this basis, the variational Bayesian model models the result statistics as a specific probability distribution and uses the distribution to contain the possibility of various changes. At the same time, the prediction period of the target wind farm is dynamically divided by combining the spatial position and real-time meteorological data. These measures can effectively analyze the correlation between wind farms, giving the variational Bayesian model the potential for generalization. We verify this generalization in Section 5.4.

## 4. Forecasting Process and Evaluation Indicators

To effectively utilize the known power fluctuations of the adjacent wind farms, we propose a new algorithm for ultra-short-term power prediction of wind farms based on the dynamic division of prediction period. Since the length of the prediction period for ultra-short-term power prediction of wind farms is fixed at 4 h, but the length of the prior information period is usually less than 4 h, this means that in the prediction period, in addition to the prior information period, there will be a non-prior information period that needs to be additionally predicted for power.

Because the length of the prior information period needs to be calculated from the wind speed and direction at each prediction moment, the length of the non-prior information period is not fixed. However, since the temporal resolution of the power is 15 min, after dividing the calculated length by 15 min and then rounding it up, the length of the non-prior information period is fixed. Therefore, we utilize TCN [21], which has a flexible structure and whose output length can be adjusted according to the needs, to train the prediction model for the non-prior information period.

**Figure 3:**
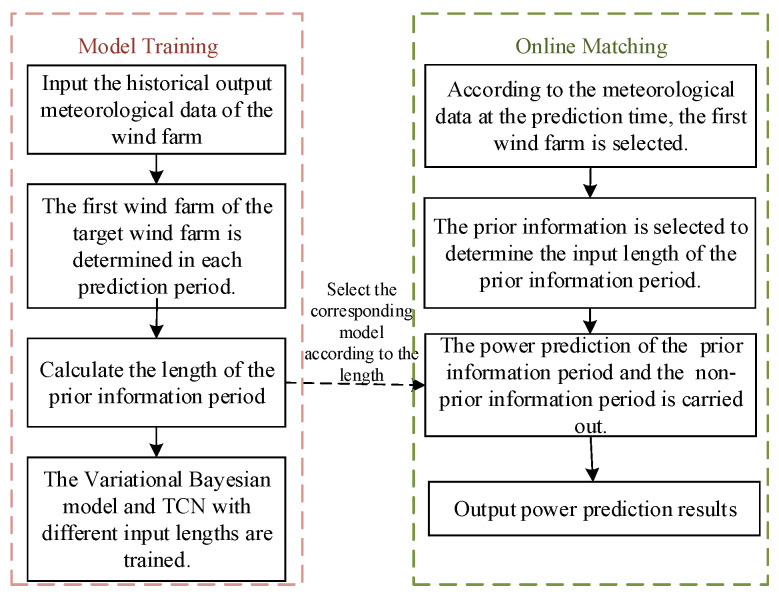
Overall prediction process diagram.

Since it is necessary to calculate the time difference between the wind farm fluctuation-related power at the prediction moment to determine the length of the prior information period and the input lengths of the models. To reduce the time consumption of each prediction and to meet the demand of ultra-short-term prediction for computing speed, we construct the variational Bayesian model and TCN model with different input lengths for the two time periods in the train set. The overall prediction process diagram is shown in Figure 3.

The specific steps are as follows:

(1) Input the relative geographical location of the target wind farm and its adjacent wind farm, as well as the measured wind speed, wind direction and power data of each wind farm.

(2) The data are divided into train set and test set. In the train set, the length of the prior information period at each prediction time is calculated, and the prediction period is classified according to different lengths.

(3) The variational Bayesian model and TCN with different input lengths are trained to predict the prior information periods and the non-prior information periods.

(4) In the test set, the input lengths of the prior information period and the non-prior information period are calculated according to the first wind farm at the prediction time. After that, the corresponding model is selected according to different lengths, and the power predictions of the prior information period and non-prior information period are carried out. Finally, the ultra-short-term power prediction result of the target wind farm for the entire prediction period is obtained.

The comparison models used in this paper are TCN, LSTM, CNN-GRU, and CNN-LSTM. For the prediction results, we use the mean absolute percentage error (MAPE) and the root mean square error (RMSE) as evaluation criteria. The specific formulas are as follows:

(14) $$R_{\mathrm{MAPE}}=\frac{1}{N}\sum_{i=1}^{N}\frac{\left|y_{i}^{\prime }-y_{i}\right|}{P_{N}}\times 100\%$$

(15) $$R_{\mathrm{RMSE}}=\sqrt{\frac{1}{N}\sum_{i=1}^{N}{\left(y_{i}^{\prime }-y\right)}^{2}}$$

where *N* is the number of predicted power points; ${y\prime }_{i}$ is the *i*-th point in the predicted power sequence; *y_i_* is the actual power value of the wind farm corresponding to the *i*-th predicted point; P_N_ is the rated capacity of the wind farm.

## 5. Case Analysis

### 5.1. Example Setup

The measured power data of three wind farms in southern China from 2022 to 2023 are used to validate the algorithm, with a time resolution of 15 min, 35,136 power data points for each wind farm, and a rated capacity of 90 MW. The dataset is divided into two parts: the train set and the test set in accordance with the ratio of 9:1. Taking FQ city as the starting point, the basic geographic location information of the three wind farms is shown in Table 1 and the relative location of each wind farm can be uniquely determined based on the relative latitude and longitude of the wind farms.

### 5.2. Example Analysis of Spatial-Temporal Correlation

To further verify that the power within *τ*_1_ of the first wind farm can provide effective prior information for the power prediction within *τ*_2_ of the target wind farm, we take the measured power data and meteorological data of two wind farms in southern China (W_1_ and W_2_) as an example for analysis. According to the measured data, it can be found that the prior information period of the wind farm is no more than two hours. Considering that the ultra-short-term prediction period of the wind farm is usually 15 min to 4 h, and the prediction period of the load is within 6 h, to make the prediction results provide a reference for maintaining the intra-day power balance, we set the ultra-short-term power prediction period to 4 h.

The measured data in W_2_ are divided into time periods of 4 h each. According to the method in Section 2.1, the time period of W_1_ as the first wind farm and W_2_ as the target wind farm is selected. The correlation between the power within *τ*_1_ and *τ*_2_ is verified by comparing the power within *τ*_12_ in the first wind farm (as shown in Figure 1).

In the *i*-th prediction period, the number of power points in the prior information period is calculated. *P_τ_*_1_ and *P_τ_*_12_ within *τ*_1_ and *τ*_12_ of the first wind farm and *P_τ_*_2_ within *τ*_2_ of the target wind farm are determined. The *P_τ_*_1,*I*_, *P_τ_*_12,*i*_, and *P_τ_*_2,*i*_ of the *i*-th prediction period are as follows:

(16) $$\begin{array}{l} P_{\tau 1,i}=[p_{1,t_{i}-h_{i}},p_{1,t_{i}-h_{i}+1},\cdots ,p_{1,t_{i}-1}]\\ P_{\tau 12,i}=[p_{1,t_{i}+1},p_{1,t_{i}+2},\cdots ,p_{1,t_{i}+h_{i}}]\\ P_{\tau 2,i}=[p_{2,t_{i}+1},p_{2,t_{i}+2},\cdots ,p_{2,t_{i}+h_{i}}] \end{array}$$

where *t_i_* is the prediction time of the *i*-th prediction period; *h_i_* is the number of power points in the prior information period of the *i*-th prediction period; $p_{1,t_{i}+h_{i}}$ is the power point at $t_{i}+h_{i}$ in W_1_; $p_{2,t_{i}+h_{i}}$ is the power point at $t_{i}+h_{i}$ in W_2_.

Five prediction periods of W_2_ are selected, and the correlation between *P_τ_*_1_ and *P_τ_*_2_, *P_τ_*_12_ and *P_τ_*_2_ is calculated by Pearson correlation coefficient. The results are shown in Table 2.

It can be seen from the table above that, compared with the correlation between *P_τ_*_12_ and *P_τ_*_2_, the correlation between *P_τ_*_1_ and *P_τ_*_2_ is greater in each prediction period. This proves the rationality of using the power of the first wind farm within *τ*_1_ as the prior information of the target wind farm and the feasibility of using the prior information to predict the power of the target wind farm in the prior information period.

### 5.3. Comparative Analysis of Forecast Results for Wind Power

#### 5.3.1. Determine the Number of Intervals of the First Wind Farm

**Figure 4:**
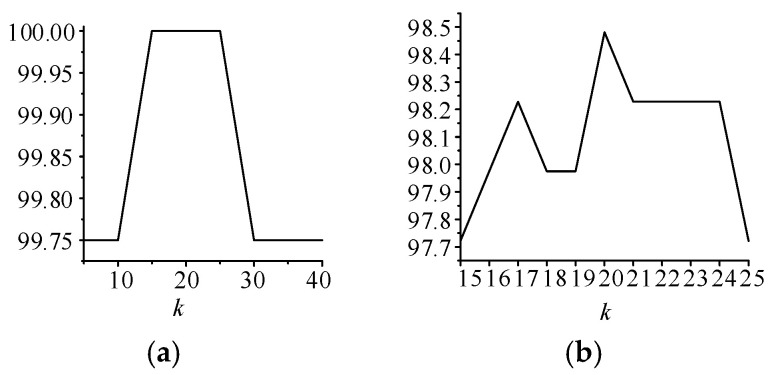
Probabilities for different values of *k*: (**a**) probability in the range of $\mu_{\Delta P_{Aft}}\pm \sigma_{\Delta P_{Aft}}$; (**b**) probability in the range of $\mu_{\Delta P_{Aft}}\pm 0.5\sigma_{\Delta P_{Aft}}$.

To obtain the mean and variance sequence sum of Δ***P_f_***, it is necessary to divide the variation sequence Δ***P′****_Fir_*. The number of intervals *k* has an important influence on the calculation of the mean and variance of the target wind farm variation Δ***P_Aft_*** in each interval. We select different values of k in the train set and divide the changes in the first wind farm into intervals by quartiles. The mean and variance sequences of the variation in the target wind farm in each interval are calculated. In the test set, the power change in the target wind farm is classified into the corresponding interval according to the prior information. The appropriate k value is selected by using the probability that the variation occurs in $\mu_{\Delta P_{Aft}}\pm \sigma_{\Delta P_{Aft}}$ and $\mu_{\Delta P_{Aft}}\pm 0.5\sigma_{\Delta P_{Aft}}$. The results are shown Figure 4.

It can be found from (a) that when the value of *k* is less than 15 or greater than 25, the variation set of the target wind farm cannot all appear in $\mu_{\Delta P_{Aft}}\pm \sigma_{\Delta P_{Aft}}$. This may be because the number of intervals will affect the number of discrete points contained in each interval, thus affecting the calculation of variance in Δ***P_f_***. Therefore, when the selection of *k* is too small or too large, the calculated variance is too large, resulting in the variation in the target wind farm not being concentrated. To further select *k* in the range of 15 to 25, so that the variation in the target wind farm is more concentrated in the vicinity, we calculate the probability of appearing in the range of $\mu_{\Delta P_{Aft}}\pm 0.5\sigma_{\Delta P_{Aft}}$. It can be found from (b) that the probability is the highest when the value of *k* is 20, so we set *k* to 20.

#### 5.3.2. Convolution Kernel Size and Number of Convolution Channels *g*

**Figure 5:**
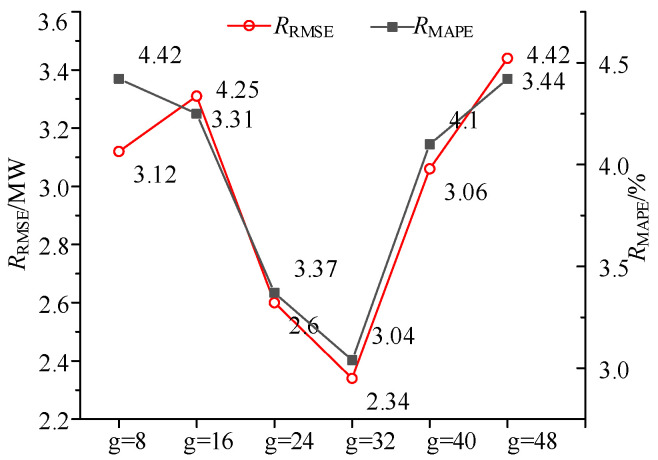
The prediction error for different values of *g*.

Due to the different number of power points to be predicted in the prior information period, the length of the input data for the variational Bayesian model is also different. The size of the convolution kernel in the convolution module is set to [1, 1]. The number *g* of convolution channels is set to different values to calculate the prediction error of the prior information period of a wind farm. The results are shown in Figure 5.

The optimization process of *g* is set to start from 8. In theory, a larger number of channels will increase the complexity and computational burden of the model, and also improve the learning ability of hidden information. However, it can be found from Figure 5 that when the number of channels increases to 32, *R*_RMSE_ gradually decreases, and the error reaches the lowest value when *g* is 32. After further increasing *g*, the error increases. It can be found that *g* has a certain influence on the performance and effect of the neural network, but it is not a simple proportional relationship. If we do not consider the characteristics of the data, the needs of the task, and other factors, blindly increasing the number of convolution channels will increase the error.

#### 5.3.3. Comparative Analysis of Forecast Results for Individual Wind Farm

**Figure 6:**
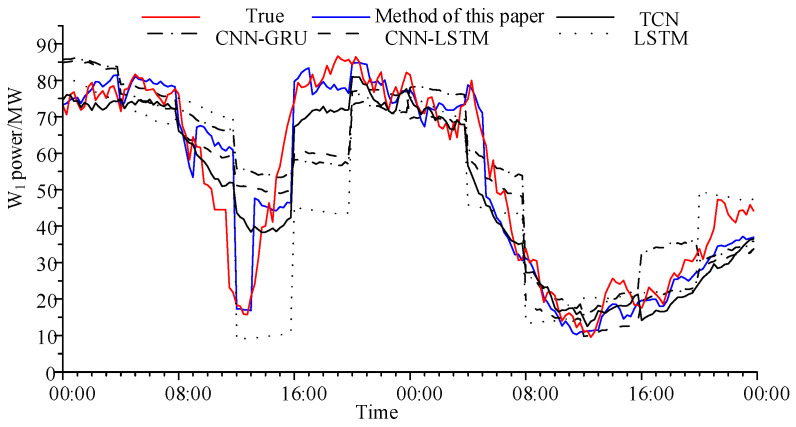
Comparison of W_1_ power prediction curves under different methods.

The prediction results of different methods for W_1_ for two days are shown in Figure 6 and the error calculation results are shown in Table 3.

It can be seen from Figure 5 and Table 3 that the power prediction effect of our method is the best. The average improvement (Imp) is used to calculate the error reduction effect of our method compared with other methods. The following conclusions can be drawn: the prediction effect of LSTM is the worst, and the Imp of *R*_MAPE_ reaches 5.25%. The prediction effect of CNN-LSTM and CNN-GRU is at least 1.9% higher than that of LSTM. The prediction effect of TCN model is about 1.25% higher than that of CNN-LSTM and CNN-GRU. However, since the number of wind farms in the example is limited, it cannot be guaranteed that the target wind farm has the first wind farm to provide effective data at each prediction time. Therefore, compared with TCN, the improvement effect of our method is not significant.

To better show the prediction effect of our method on wind power under fluctuating conditions, the peak–valley difference is greater than 0.1 P_N_ and the first wind farm that can be found at the prediction time is taken as the index. Five prediction cycles with large fluctuations in wind power in the test set are selected for prediction. The average prediction error of each model is shown in Table 4.

**Figure 7:**
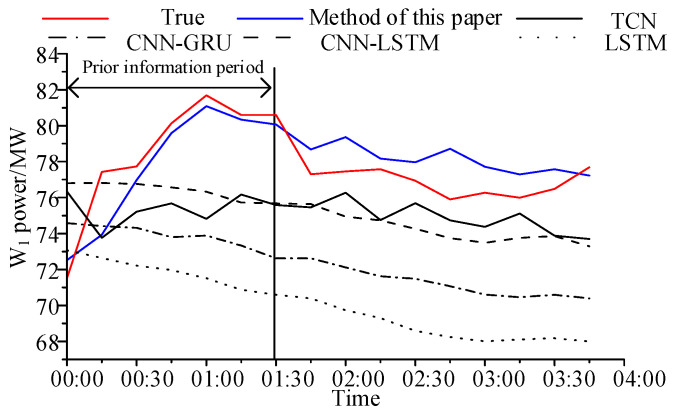
Comparison of predicted power curves of W_1_ under different methods (h = 6).

It can be seen from Table 4 that the improvement effect of our method is more obvious when the wind power fluctuates greatly, and the target wind farm can find the first wind farm at the prediction time. Compared with Table 3, the Imp of *R*_MAPE_ for TCN is increased by 4%. Taking a period with large fluctuations as an example, the prediction curves of different methods are shown in Figure 7.

It can be seen from Figure 6 that in the prior information period, LSTM only learns the time series features and has the worst prediction effect. After adding the CNN kernel that captures spatial features, the prediction effects of CNN-LSTM and CNN-GRU improve. However, it is still impossible to learn the fluctuations in the wind farm. TCN with multiple convolutional layers can learn the characteristics of different time scales and better fit the fluctuations of wind farms. However, the spatial and temporal correlation between wind farms is time-varying, and it still cannot adapt to a sudden change in power. Our method is not only to learn the historical power of the target wind farm, but also to directly use the power fluctuations that have occurred in the first wind farm as prior information to predict the power of the target wind farm in the period with prior information, which can reflect the sudden change in power caused by the change in wind speed in time. Therefore, it can reflect the sudden change in power caused by wind speed change in time.

#### 5.3.4. Comparative Analysis of Forecast Results for Multiple Wind Farms

**Figure 8:**
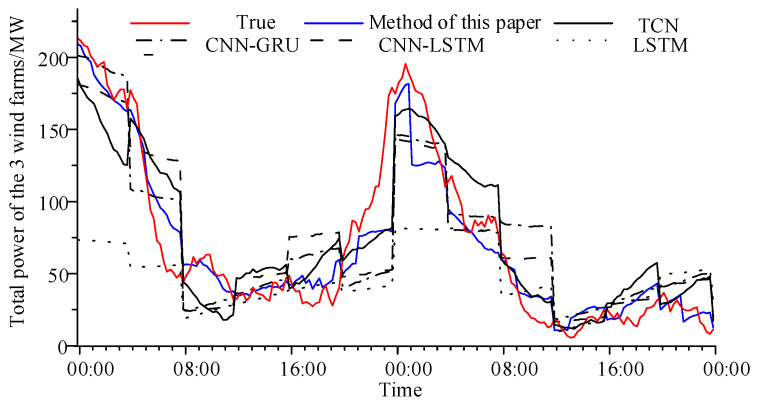
Comparison of the total power prediction curves of multiple wind farms under different methods.

The total power of the three adjacent wind farms was predicted using a rolling approach over a two-day period. Figure 8 shows the rolling prediction curves from the different methods, with a 4 h prediction cycle repeated multiple times during the two days. The prediction errors are shown in Table 5.

Compared with Table 3, it can be seen that the prediction error of the total power of multiple wind farms by most methods is lower than that of a single wind farm. Among them, the prediction effect of our method still maintains strong robustness. The Imp for the other methods decreased compared to Table 3. The reason may be that the power fluctuations in multiple wind farms are not synchronized, and when they are integrated into a single power curve, they cancel each other out and the fluctuations are smoothed down. In addition, for multiple wind farms, not all of the wind farms have a first wind farm that can provide valid prior information about the changes in their forecasts. So, the improvement in our methodology is evenly distributed to the forecasts of the remaining wind farms.

### 5.4. Verification of Generalizability of Variational Bayesian Model

**Figure 9:**
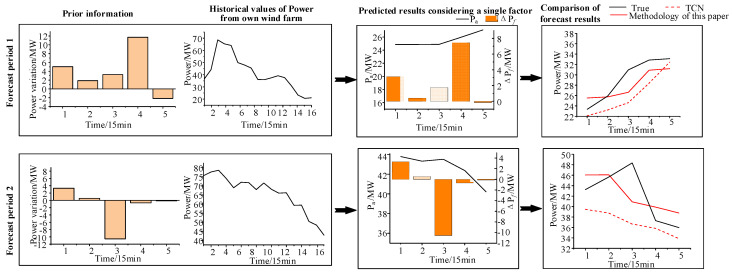
Prediction effect of time difference period for unseen scenes.

We verify the generalizability of the model by its prediction effect on unseen scenarios. In the train set and test set, the following designs were carried out: (1) In the train set, select the samples such that W_1_ is the first wind farm for W_2_ and exclude samples for which the wind direction is 193°~213° W. (2) In the test set, the samples with wind directions from 193° and 213° are selected for prediction. The prediction effect of the model is shown in Figure 9.

Figure 9 shows the comparison of the prediction results for the two prediction periods of the test set: the prior information; the historical power data of the target wind farm; the prediction results ***P_a_*** and Δ***P_f_*** only considering a single influencing factor; comparison of final prediction results in prior information period. It can be seen that, for scenarios that have not occurred, TCN cannot accurately determine the degree of contribution of adjacent wind farm, whereas the variational Bayesian model can still obtain the power prediction curve of the prior information period according to the prior information provided by the first wind farm and the historical data of the target wind farm. The prediction effect of our method is significantly better than that of TCN, which effectively proves the interpretability and generalizability of the variational Bayesian model.

## 6. Conclusions

We propose a new algorithm for the ultra-short-term prediction of wind farm power, considering the wind speed fluctuation in adjacent wind farms and dynamically dividing the period with or without prior information. Comparing the actual data with other methods, we draw the following conclusions:

(1) By using the relative geographical location between wind farms and real-time wind speed and direction, each prediction period of the target wind farm is dynamically divided. The prior information provided by the first wind farm is applied to the power prediction of the target wind farm during the prior information period, which can timelier reflect the power fluctuations caused by wind speed changes.

(2) The variational Bayesian model can effectively fit the relationship between the prediction results of a single influencing factor and the actual power sequence. Compared with other models, it is more interpretable and generalizable and can improve the accuracy of power prediction.

(3) In the case of adding adjacent wind farms, the variational Bayesian model does not need to be changed. It only needs to use the geographical location of the new wind farm to add it to the selection of the first wind farm, which has good engineering practicability.

(4) When different wind farms are considered first, different terrains between wind farms will lead to differences in the influence of prior information. Since the wind farms we use are in a relatively flat geographical location, this factor is not considered. The next step will be to study how to add this factor to the model to further improve its prediction accuracy.

## Figures and Tables

**Table 1:** Basic geographic location information of wind farm.

Wind Farm	Relative Longitude	Relative Latitude
W_1_	0.51° E	0.20° N
W_2_	0.33° E	0.15° N
W_3_	0.30° E	0.16° N

**Table 2:** The correlation between wind farm power sequences in different prediction periods.

Forecast Period	1	2	3	4	5
*P_τ_*_1_ and *P_τ_*_2_	0.8841	0.8627	0.8534	0.8954	0.9237
*P_τ_*_12_ and *P_τ_*_2_	0.8297	0.8052	0.8131	0.7904	0.9086

**Table 3:** Comparison of W_1_ power prediction error under different methods.

Different Methods	*R*_MAPE_		*R*_RMSE_	
	Average/%	Imp	Average/MW	Imp
LSTM	11.52	5.25	14.04	6.84
CNN-GRU	8.82	2.54	11.32	4.12
CNN-LSTM	8.80	2.52	10.83	3.63
TCN	7.56	1.28	9.54	2.34
Method of this paper	6.28	-	7.20	-

**Table 4:** Comparison of prediction errors of different methods when the output fluctuates greatly.

Different Methods	*R*_MAPE_		*R*_RMSE_	
	Average/%	Imp	Average/MW	Imp
LSTM	12.56	10.23	14.71	12.04
CNN-GRU	10.69	8.36	12.01	9.34
CNN-LSTM	10.45	8.12	11.96	9.29
TCN	7.61	5.28	8.02	5.35
Method of this paper	2.33	-	2.67	-

**Table 5:** Comparison of total power prediction errors of multiple wind farms under different methods.

Different Methods	*R*_MAPE_		*R*_RMSE_	
	Average/%	Imp	Average/MW	Imp
LSTM	10.59	4.87	44.93	21.98
CNN-GRU	8.24	2.52	31.48	8.53
CNN-LSTM	8.10	2.38	31.07	8.12
TCN	6.85	1.13	25.68	2.73
Method of this paper	5.72	-	22.95	-

## Data Availability

Data are contained within the article.
